# The effect of enteral bolus feeding on regional intestinal oxygen saturation in preterm infants is age-dependent: a longitudinal observational study

**DOI:** 10.1186/s12887-019-1805-z

**Published:** 2019-11-04

**Authors:** Sara J. Kuik, Anne G. J. F. van Zoonen, Arend F. Bos, Koenraad N. J. A. Van Braeckel, Jan B. F. Hulscher, Elisabeth M. W. Kooi

**Affiliations:** 1 University of Groningen, University Medical Center Groningen, Beatrix Children’s Hospital, Division of Neonatology, Groningen, the Netherlands; 2 University of Groningen, University Medical Center Groningen, Department of Surgery, Division of Pediatric Surgery, Groningen, the Netherlands

**Keywords:** Cerebral oxygen saturation, Enteral feeding, Feeding volumes, Fractional tissue oxygen extraction, Intestinal oxygen saturation, Postnatal age, Postmenstrual age

## Abstract

**Background:**

The factors that determine the effect of enteral feeding on intestinal perfusion after preterm birth remain largely unknown. We aimed to determine the effect of enteral feeding on intestinal oxygen saturation (r_int_SO_2_) in preterm infants and evaluated whether this effect depended on postnatal age (PNA), postmenstrual age (PMA), and/or feeding volumes. We also evaluated whether changes in postprandial r_int_SO_2_ affected cerebral oxygen saturation (r_c_SO_2_).

**Methods:**

In a longitudinal observational pilot study using near-infrared spectroscopy we measured r_int_SO_2_ and r_c_SO_2_ continuously for two hours on postnatal Days 2 to 5, 8, 15, 22, 29, and 36. We compared preprandial with postprandial values over time using multi-level analyses. To assess the effect of PNA, PMA, and feeding volumes, we performed Wilcoxon signed-rank tests or logistic regression analyses. To evaluate the effect on r_c_SO_2_, we also used logistic regression analyses.

**Results:**

We included 29 infants: median (range) gestational age 28.1 weeks (25.1–30.7) and birth weight 1025 g (580–1495). On Day 5, r_int_SO_2_ values decreased postprandially: mean (SE) 44% (10) versus 35% (7), *P* = .01. On Day 29, r_int_SO_2_ values increased: 44% (11) versus 54% (7), *P* = .01. Infants with a PMA ≥ 32 weeks showed a r_int_SO_2_ increase after feeding (37% versus 51%, *P* = .04) whereas infants with a PMA < 32 weeks did not. Feeding volumes were associated with an increased postprandial r_int_SO_2_ (per 10 mL/kg: OR 1.63, 95% CI, 1.02–2.59). We did not find an effect on r_c_SO_2_ when r_int_SO_2_ increased postprandially.

**Conclusions:**

Our study suggests that postprandial r_int_SO_2_ increases in preterm infants only from the fifth week after birth, particularly at PMA ≥ 32 weeks when greater volumes of enteral feeding are tolerated. We speculate that at young gestational and postmenstrual ages preterm infants are still unable to increase intestinal oxygen saturation after feeding, which might be essential to meet metabolic demands.

**Trial registration:**

For this prospective longitudinal pilot study we derived patients from a larger observational cohort study: CALIFORNIA-Trial, Dutch Trial Registry NTR4153.

## Background

Introducing preterm infants to enteral feeding is challenging. Gastrointestinal (GI) motility of preterm infants is limited causing delay in gastric emptying and intestinal transit. This in turn could easily result in intolerance to feeding [1]. Enteral feeding has beneficial effects on the structural and functional development of the GI tract [1, 2]. The passage of enteral feeds leads to an increased metabolic demand on the small intestine. This results in increased intestinal perfusion from the superior mesenteric artery (SMA) known as postprandial hyperaemia [3, 4]. If this increased metabolic demand after enteral feeding cannot be met, feeding intolerance (FI) may occur, resulting in delayed full enteral feeding (FEF) and possibly even necrotizing enterocolitis (NEC) [5–8]. Furthermore, as preterm infants are at risk of impaired cerebrovascular autoregulation, postprandial redistribution of blood in favour of the intestines may result in cerebral underperfusion [9–11].

Near-infrared spectroscopy (NIRS) is a non-invasive method to assess end-organ perfusion in preterm infants [8, 12–14]. It allows us to measure regional tissue oxygen saturation (rSO_2_) continuously [12–14]. From this measure fractional tissue oxygen extraction (FTOE) can be calculated, which reflects the balance between oxygen delivery and consumption [12–14].

Recent studies on NIRS or Doppler flow measurements of the SMA reported that healthy preterm infants, who tolerate enteral feeding of at least 100 mL/kg/day, demonstrate increased intestinal postprandial perfusion while cerebral perfusion remains stable [2, 15–17]. Nevertheless, little is known about whether this capability of the premature intestine to increase its perfusion after feeding is dependent on postnatal age (PNA), postmenstrual age (PMA), and/or feeding volumes. In addition, it remains unclear if cerebral perfusion also remains stable when postprandial redistribution of blood in favour of the intestines occurs soon after birth or in younger infants. Furthermore, studies that evaluated whether the presence or absence of postprandial intestinal hyperaemia is associated with the development of FI or with the development of NEC, are limited. Therefore our aim was to determine the effect of enteral bolus feeding on intestinal oxygen saturation (r_int_SO_2_) and extraction in preterm infants during the first five weeks after birth, and to evaluate whether this effect depended on PNA, PMA, and/or feeding volumes. Furthermore, we explored whether the cerebral oxygen saturation (r_c_SO_2_) and extraction changed when postprandial r_int_SO_2_ increased after enteral feeding.

## Methods

### Participants

For this prospective, longitudinal, observational, exploratory study we derived patients from a larger observational cohort study at our tertiary referral neonatal intensive care unit (NICU) that aimed to identify prognostic markers for the development of NEC in high-risk neonates (CALIFORNIA-Trial, Dutch Trial Registry NTR4153) [18, 19]. For this trial, all infants who were at high risk of developing NEC, who were born between October 2012 and February 2014, and had been admitted to our NICU were eligible for inclusion. High-risk infants were defined as infants with a gestational age (GA) of less than 30 weeks *or* a birth weight (BW) of less than 1000 g, or a GA of less than 32 weeks *and* a BW below 1200 g, or preterm-born infants who had been exposed to indomethacin antenatally [20]. Exclusion criteria were congenital abdominal malformations or large chromosomal defects. For this pilot sub-study, which was part of a new scientific project, we started with precisely recording the feeding times from August 2013 onwards and included all preterm infants born between August 2013 and January 2014 and who had been admitted to our NICU. All infants were included after their parents had given written informed consent within 72 h after birth. The study was approved by the ethical review board of University Medical Center Groningen.

### Feeding data

All infants received enteral feeding through nasogastric tubes. Feedings consisted of preterm formula, mother’s own milk, donor mother’s milk, or a combination. Infants who weighed less than 1200 g received enteral bolus feeding every two hours for 10 to 15 min by tube and open syringe using gravity. Infants who weighed more than 1200 g were fed once every three hours. As feeding volumes are relatively larger in case of bolus feeding once every three hours than once every two hours, we recorded feeding volumes in mL/kg/day but also in mL/kg during the NIRS measurement. All infants received 10 to 20 mL/kg on the first day after birth. Subsequently, feeding volumes were increased daily by 20 mL/kg/day unless gastrointestinal problems, such as recurrent vomiting or gastric retentions exceeding 5 mL, occurred repeatedly.

The feeding times were recorded during the NIRS measurements. We recorded the time at which feeding commenced, that is the time the feeding bolus was connected to the feeding tube, and the time feeding ended, that is the time the feeding tube was empty, feeding volumes (expressed in mL), and the type of feeding received by the infant.

### Gastrointestinal complications

We recorded whether infants developed FI, NEC or a spontaneous intestinal (SIP) perforation. FI was defined as > 50% decrease in ml/kg/day of enteral feeding or withdrawal of enteral feeding because of abdominal distension, vomiting, abundant gastric retentions, bilious or bloody gastric retentions, or bloody stools.

### Clinical characteristics

Prospectively, we collected data on GA, BW, PMA, PNA, sex, Apgar scores, SNAPPE-II score as measure for severity of illness [21], respiratory support, PCO_2_, pH, haemoglobin, systolic, diastolic, and mean arterial blood pressure, the need for fluid resuscitation or inotropic support, the presence of a hemodynamically significant patent ductus arteriosus (PDA), and the presence of cerebral pathologies on cerebral ultrasound.

### Near-infrared spectroscopy

We used the INVOS 5100C near-infrared spectrometer in combination with neonatal SomaSensors (Medtronic, Dublin, Ireland) to measure r_int_SO_2_ and r_c_SO_2_. We used Mepitel® film (Mölnlycke, Sweden), which does not adversely affect INVOS integrity or validity [22], to keep the sensor in place and as a skin barrier below each sensor. To measure r_int_SO_2_ we placed the sensor infraumbilically on the central abdomen. To measure r_c_SO_2_ we placed the sensor on the left or right frontoparietal side of the head. Intestinal and cerebral rSO_2_ were measured for two uninterrupted hours, starting at 5 min prior to feeding, during postnatal Days 2 to 5, 8, 15, 22, 29, and 36. The study ended prior to Day 36 if an infant developed NEC Bell Stage ≥2, died, or was discharged from the NICU. We removed artefacts from the rSO_2_ measurements. Artefacts were defined as instances recorded as sensor displacement, or a sudden major non-physiologic increase or decrease of the rSO_2_ values within seconds, which suggests an incorrect measurement. We measured transcutaneous arterial oxygen saturation (SpO_2_) simultaneously with the rSO_2_ measurements using Nellcor (Medtronic) sensors. Next, we calculated intestinal and cerebral FTOE with the following formula: (SpO_2_-rSO_2_)/SpO_2_. The FTOE reflects the balance between oxygen delivery to the tissue measured and oxygen consumption of the tissue measured, and depends less on changes in arterial oxygen saturation [10].

### Statistical analyses and sample size

For statistical analyses we used SPSS 23.0 (IBM Corp., Armonk, NY, USA). We described the patient characteristics in terms of median (range) values. First, after confirming normal distribution of the data, we calculated the mean and standard error of the mean (SE) of all NIRS measurements at three points in time on postnatal Days 2 to 5, 8, 15, 22, 29, and 36, viz. 5 min prior to feeding and 10 to 30 min and 30 to 60 min after feeding had commenced. SE was preferred over standard deviation, given the comparison of means and given the small sample size, which may hamper accurate estimation of the means [23, 24]**.** Next, we built a multi-level model for each dependent variable using the statistical program MLwiN 2.15 (University of Bristol, Bristol, UK) [25]. Given the presence of missing data, an advantage of multilevel analysis is that this analysis calculates weighted means and their standard errors, which takes the number of data points per infant into account, thus allowing infants with more data points to weigh more into the estimated mean than infants with less data points. Four models, one for each dependent variable (r_int_SO_2_, r_c_SO_2_, intFTOE, and cFTOE) were specified with measurements (Level 1) nested within subjects (Level 2). Thus, the dependency between measurements was taken into consideration in which the intercept represented the baseline measurement (before feeding) on Day 2. To compare preprandial measurements with measurements 10 to 30 and 30 to 60 min postprandially, each model consisted of 27 terms (9 days multiplied by the three points in time; that is each term is defined as one measurement of one day). A *t* test was used to test for differences between an estimated mean and the intercept [26]. We tested the contrast of the sum of parameters from which each estimate is derived using a chi-square test with one degree of freedom to test for differences between two estimated means.

Second, to evaluate whether the effect of enteral bolus feeding on the r_int_SO_2_ depended on PMA, we clustered the measurements into different groups, that is PMA < or ≥ 30 weeks and < or ≥ 32 weeks and performed a Wilcoxon signed rank test between preprandial and postprandial r_int_SO_2_ values. Next, to determine whether feeding volumes were associated with the effect of enteral feeding on r_int_SO_2_, we used a univariate logistics regression analysis between postprandial r_int_SO_2_ values (categorized into increase or no increase) and the amount of the bolus enteral feeding per 10 mL/kg.

Thereafter, to explore whether a postprandial r_int_SO_2_ increase was associated with a decreased postprandial r_c_SO_2_, we performed a logistic regression analysis between categorized data; that is increase or no increase of the r_int_SO_2_ versus decrease or no decrease of the r_c_SO_2_. Finally, we performed a subanalysis between infants who did and did not develop any GI complications. Infants were categorized into four groups; Uncomplicated, FI, NEC, and SIP. As two out of the three NEC infants developed NEC within 14 days, we clustered the data from the first two postnatal weeks and calculated delta’s between baseline r_int_SO_2_ and postprandial r_int_SO_2_ values, and performed a Mann Whitney U between delta’s of the infants with and without a GI complication. For this subanalysis, we used a non-parametric test as the delta’s in this small sample size were not normally distributed and therefore presented these data in medians [IQRs].

Throughout the analyses a *P* value < .05 was considered statistically significant. We chose not to correct for multiple testing in this explorative study.

## Results

### Patient characteristics

**Fig. 1:**
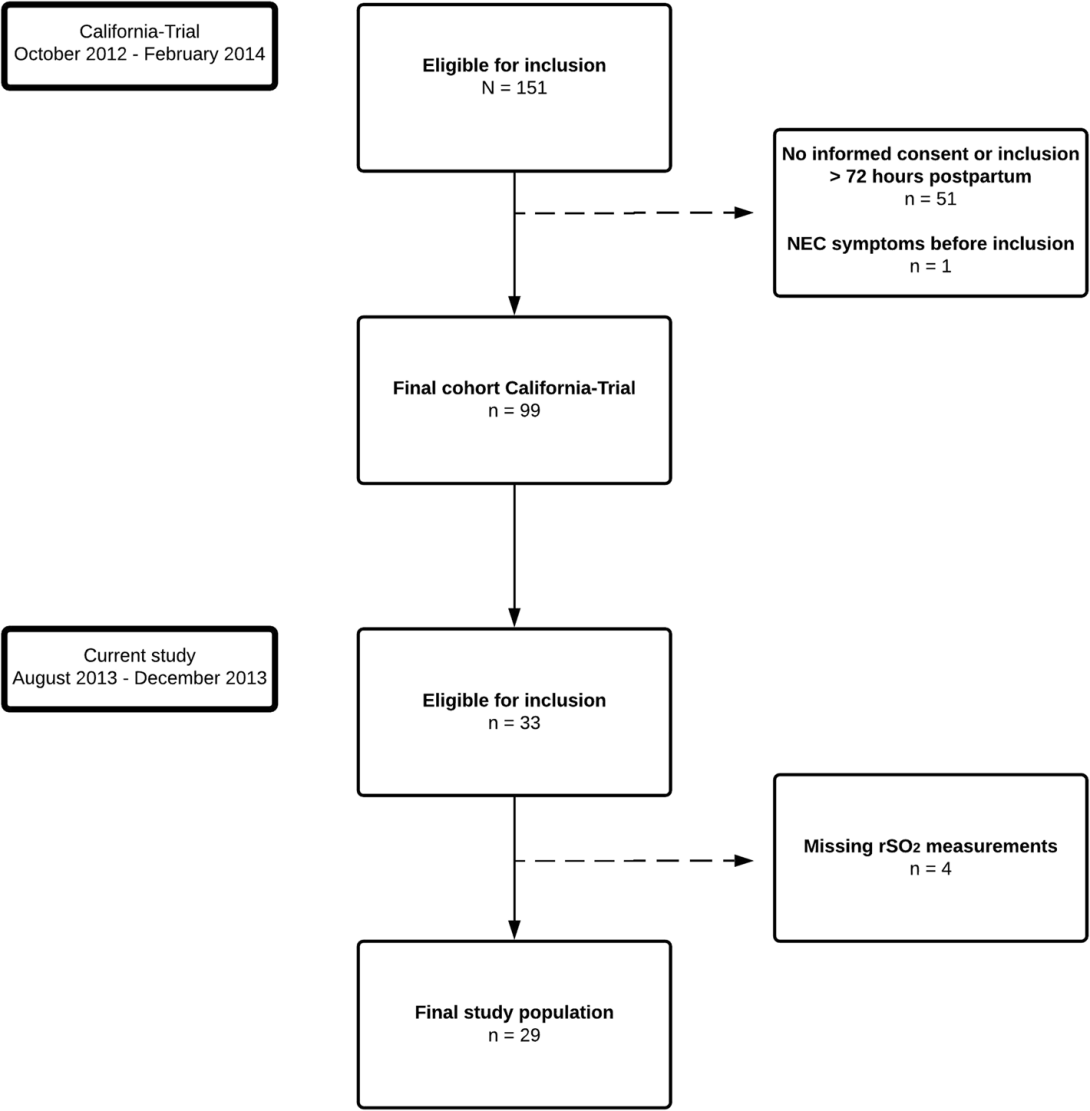
Flow diagram of the study population

We included 29 patients out of 33 eligible patients (Fig. 1). We had to exclude four infants because of missing rSO_2_ data. The 29 remaining infants had a median GA of 28.1 weeks (range 25.1–30.7) and a median BW of 1025 g (range 580–1495). Table 1 provides an overview of the patient characteristics. Three infants died during the study period after a median of 21 days (range 16–25) after birth: one infant died of NEC, one of multi-organ failure as a result of sepsis, and one infant died of progressive respiratory failure. Three infants developed NEC Bell’s Stage ≥2 on postnatal Days 7, 10, and 30, respectively. Two infants developed a spontaneous intestinal perforation on postnatal Day 8 and Day 12. Thirteen patients were discharged from the NICU prior to the 36th day (from Day 15 onward). In 16 patients we were unable to measure intestinal NIRS during the first two to eight days after birth because of the placement of umbilical catheters taped to the infraumbilical skin or as a result of a lack of space on the infants’ abdomens.

**Table 1:** Patient characteristics during the study period

Study population	*n* = 29
Boys/Girls	16/13 (65%/45%)
Gestational age, weeks	28 + 1 (25 + 1–30 + 5)
Birth weight, g	1025 (580–1495)
Sets of twins	4 (14%)
Small-for-gestational-age (*P* < 10)	6 (21%)
Head circumference on day of birth, centimetres	25.0 (22.5–29.0)
Apgar score at 5 min	7 (2–9)
SNAPPE-II score	28 (0–77)
Intestinal pathologies	
Necrotizing enterocolitis/spontaneous intestinal perforation	4 (14%)
Sepsis (including suspected sepsis)	22 (76%)
Circulatory failure	
Fluid resuscitation	7 (24%)
Inotropic treatment	2 (7%)
Respiratory support^a^	
Mechanical ventilation	16 (55%)
Continuous positive airway pressure	27 (93%)
High flow	7 (24%)
Low flow or no support	15 (52%)
Cerebral lesions	
Germinal matrix haemorrhage-intraventricular haemorrhage	
Grade I	6 (21%)
Grade II	2 (7%)
Transient periventricular echodensities	10 (34%)
Periventricular leukomalacia	13 (45%)
Patent ductus arteriosus	
Expectative policy	7 (24%)
Ibuprofen treatment	6 (21%)
Surgical clip	3 (10%)
Hyperbilirubinemia	23 (79%)
Anaemia	19 (66%)
Hemoglobin (mmol/L)	
Day 2	9.1 (7.6–11.6)
Day 3	9.0 (6.5–11.6)
Day 4	8.6 (6.5–11.9)
Day 5	8.4 (6.8–11.9)
Day 8	8.5 (6.9–10.6)
Day 15	8.2 (6.2–9.7)
Day 22	8.0 (5.7–8.4)
Day 29	7.8 (6.2–9.5)
Day 36	8.3 (6.4–9.8)
Enteral feeding^a^	
Mother’s milk	24 (83%)
Preterm formula	20 (69%)
Donor mother’s milk	10 (34%)
Infusion rate bolus feeding (mL/min)	3.4 (0.1–60.0)

Abbreviations: SD, standard deviation. SNAPPE-II, Score for Neonatal Acute Physiology - Perinatal Extension II. The data are expressed as median (range) or as numbers (percentages) unless otherwise specified. ^a^The numbers exceed totals, because a single infant could have several respiratory supports and several types of enteral feeding during the first 36 days after birth

### The effect of feeding on intestinal oxygenation in relation to postnatal age

**Fig. 2:**
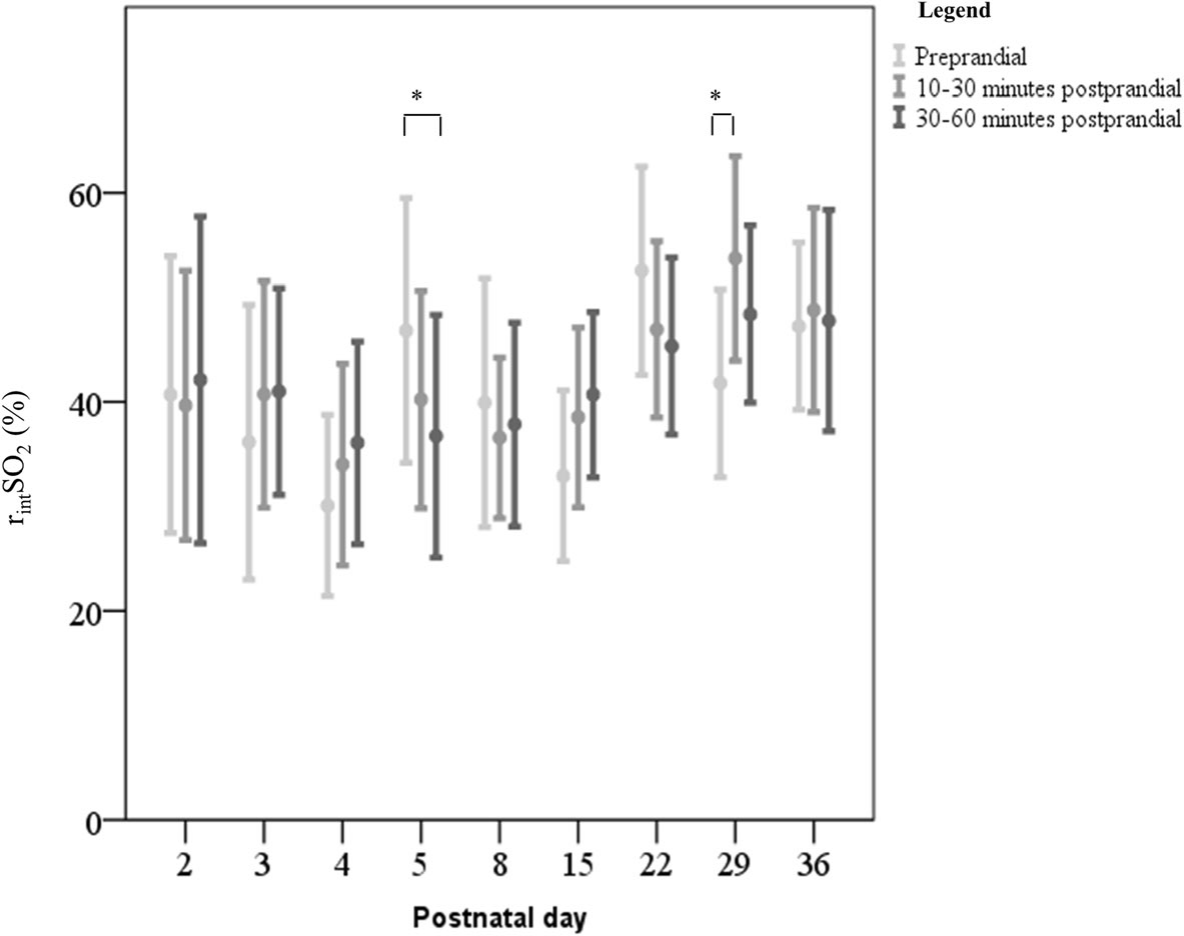
Preprandial r_int_SO_2_ values compared to postprandial r_int_SO_2_ values on postnatal days The bars represent the mean and standard error of the mean of individual r_int_SO_2_ values before and after enteral feeding. The mean r_int_SO_2_ is marked with a o within the bars. Statistically significant differences are marked with an asterisk: * < .05

On Day 5, mean postprandial r_int_SO_2_ values were lower than mean preprandial values: 10 to 30 min after feeding r_int_SO_2_ was 38% (SE 7) versus 44% (SE 10) before feeding, just failing to reach significance (*n* = 12, *P* = .07), while 30 to 60 min after feeding the decrease was significant (35%, SE 7, versus 44%, SE 10, n = 12, *P* = .01). On Day 29 (median postmenstrual age: 31.7 weeks, range 29.3–34.7), mean postprandial r_int_SO_2_ values 10 to 30 min after feeding increased with respect to preprandial values (r_int_SO_2_ 54%, SE 7, versus 44%, SE 11, *n* = 10, *P* = .01). The intFTOE did not change concomitantly. We provide a complete overview of the results in Table 2 and Fig. 2.

**Table 2:** Preprandial compared to postprandial values of r_int_SO_2_, r_c_SO_2_, intFTOE, and cFTOE values on postnatal days

	M1 Mean (SE)	M2 Mean (SE)	M3 Mean (SE)	*P* value M1 vs. M2	*P* value M1 vs. M3
Day 2					
r_int_SO_2_ (%, *n* = 10)	40 (11)	38 (7)	40 (7)	.46	1.00
r_c_SO_2_ (%, *n* = 28)	77 (4)	77 (3)	78 (3)	.71	.43
intFTOE (*n* = 9)	0.48 (0.14)	0.57 (0.14)	0.47 (0.14)	.24	.85
cFTOE (*n* = 27)	0.13 (0.04)	0.14 (0.04)	0.13 (0.04)	.97	.90
Day 3					
r_int_SO_2_ (%, *n* = 7)	37 (11)	39 (7)	41 (7)	.58	.32
r_c_SO_2_ (%, *n* = 25)	75 (4)	76 (3)	76 (3)	.30	.37
intFTOE (*n* = 7)	0.58 (0.14)	0.48 (0.14)	0.51 (0.14)	.18	.31
cFTOE (n = 25)	0.17 (0.04)	0.17 (0.04)	0.16 (0.04)	.80	.46
Day 4					
r_int_SO_2_ (%, *n* = 11)	34 (10)	34 (7)	35 (7)	.91	.59
r_c_SO_2_ (%, n = 28)	73 (4)	72 (3)	73 (3)	.48	.83
intFTOE (*n* = 11)	0.65 (0.14)	0.59 (0.14)	0.57 (0.14)	.34	.19
cFTOE (*n * = 28)	0.18 (0.04)	0.20 (0.04)	0.17 (0.04)	.27	.67
Day 5					
r_int_SO_2_ (%, *n* = 12)	44 (10)	38 (7)	35 (7)	.07	.01*
r_c_SO_2_ (%, *n* = 27)	73 (4)	72 (3)	72 (3)	.32	.19
intFTOE (*n* = 12)	0.49(0.14)	0.58(0.14)	0.48 (0.14)	.16	.84
cFTOE (*n* = 27)	0.19 (0.04)	0.21 (0.04)	0.21 (0.04)	.26	.27
Day 8					
r_int_SO_2_ (%, *n* = 12)	39 (10)	38 (7)	35 (7)	.61	.21
r_c_SO_2_ (%, *n * = 25)	67 (4)	71 (3)	73 (3)	.01*	<.01*
intFTOE (*n * = 12)	0.59 (0.13)	0.56 (0.13)	0.62 (0.14)	.63	.66
cFTOE (n = 25)	0.25 (0.04)	0.22 (0.04)	0.20 (0.04)	.27	.03*
Day 15					
r_int_SO_2_ (%, *n* = 15)	34 (10)	39 (7)	38 (7)	.13	.15
r_c_SO_2_ (%, *n* = 19)	66 (4)	64 (3)	63 (3)	.22	.09
intFTOE (n = 15)	0.59 (0.13)	0.51 (0.13)	0.58 (0.13)	.17	.87
cFTOE (n = 19)	0.28 (0.05)	0.26 (0.04)	0.27 (0.04)	.33	.76
Day 22					
r_int_SO_2_ (%, n = 11)	48 (10)	47 (7)	45 (7)	.67	.35
r_c_SO_2_ (%, *n* = 14)	57 (4)	58 (3)	58 (3)	.46	.39
intFTOE (n = 11)	0.46 (0.14)	0.45 (0.14)	0.46 (0.14)	.93	.90
cFTOE (n = 14)	0.36 (0.05)	0.27 (0.05)	0.33 (0.04)	<.01*	.23
Day 29					
r_int_SO_2_ (%, n = 10)	44 (11)	54 (7)	50 (7)	.01*	.18
r_c_SO_2_ (%, n = 12)	62 (5)	63 (3)	62 (3)	.63	.87
intFTOE (n = 10)	0.43 (0.14)	0.40 (0.14)	0.45 (0.14)	.65	.78
cFTOE (n = 12)	0.26 (0.05)	0.27 (0.05)	0.28 (0.05)	.71	.42
Day 36					
r_int_SO_2_ (%, *n* = 8)	47 (11)	49 (7)	46 (7)	.56	.84
r_c_SO_2_ (%, n = 8)	65 (5)	65 (3)	66 (3)	.80	.52
intFTOE (n = 8)	0.40 (0.14)	0.37 (0.14)	0.50 (0.14)	.65	.21
cFTOE (n = 8)	0.26 (0.05)	0.26 (0.05)	0.23 (0.05)	.88	.37

Abbreviations: *M1* Measurement 1 (preprandial), *M2* Measurement 2 (10 to 30 min postprandial), *M3* Measurement 3 (30 to 60 min postprandial). The data are expressed as mean (standard errors of the mean) unless otherwise specified. * = *P* value < .05

### The effect of feeding on r_int_SO_2_ in relation to postmenstrual age

**Fig. 3:**
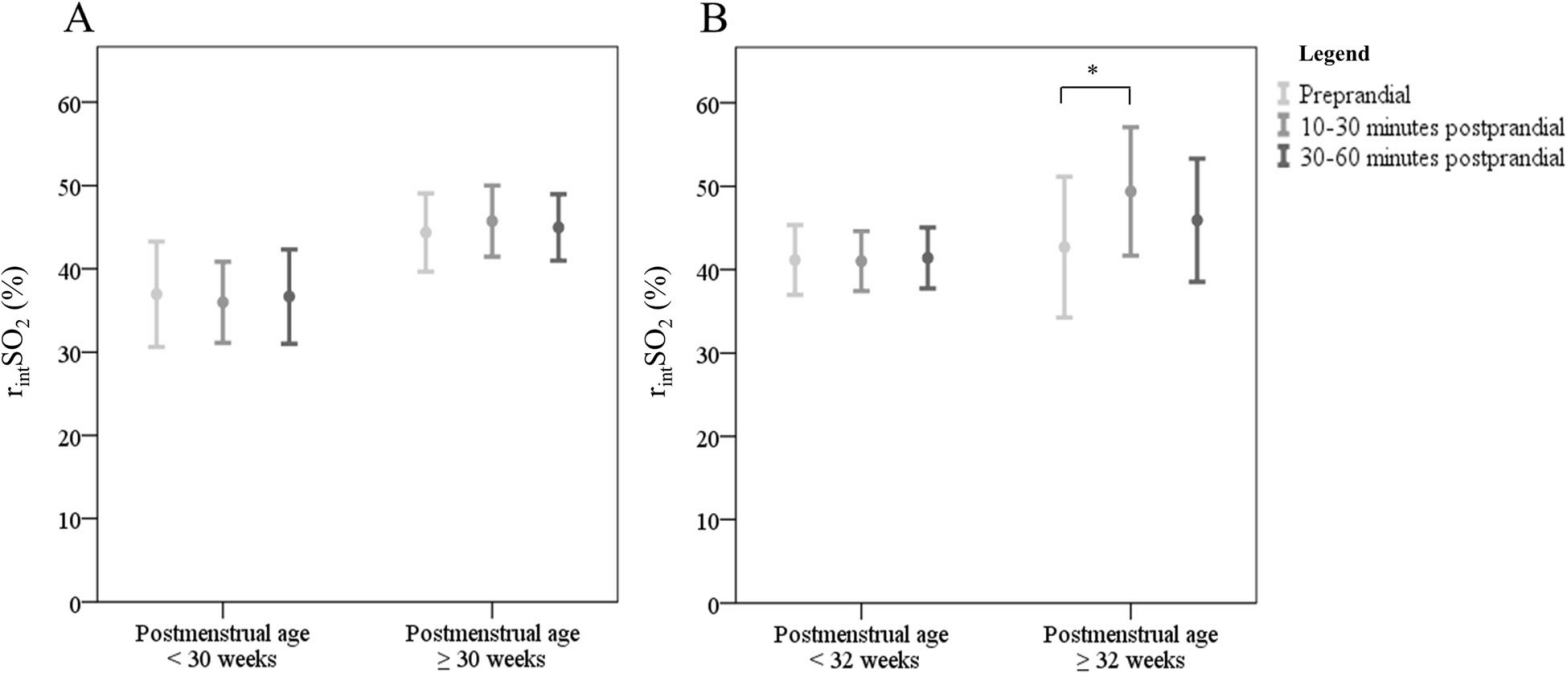
Preprandial r_int_SO_2_ values compared to postprandial values between PMA groups The bars represent the mean and standard error of the mean of individual r_int_SO_2_ values before and after enteral feeding for the different PMA groups; PMA < or ≥ 30 weeks (**a**), PMA < or ≥ 32 weeks (**b**). The mean r_int_SO_2_ is marked with a o within the bars. Statistically significant differences are marked with an asterisk: * < .05

We found that infants with a PMA ≥ 32 weeks showed a significant postprandial increase of the r_int_SO_2_ 10 to 30 min after feeding (37% versus 51%, *P* = .04, n = 10, 13 measurements) and a non-significant increase 30 to 60 min after feeding (37% versus 44%, *P* = .06, *n* = 10, 13 measurements). All data are presented in Fig. 3.

### The effect of enteral bolus feeding on r_int_SO_2_ in relation to feeding volumes

We found a significant association between feeding volumes (mL/kg) and the change in r_int_SO_2_ 10 to 30 min after feeding. For every 10 mL/kg more enteral feeding per bolus 10 to 30 min after feeding, the odds score for an increasing postprandial r_int_SO_2_ was 1.6 times higher (95% CI, 1.02–2.59, *P* = .04). Feeding volumes were not significantly associated with the change in r_int_SO_2_ 30 to 60 min after feeding. Table 3 provides an overview of enteral feeding volumes.

**Table 3:** Enteral feeding volumes of all included infants per day and during NIRS measurement

Day	Feeding mL/kg/day	Feeding mL/kg/measurement
2 (n = 29)	20.8 (17.8–26.7)	2.1 (1.4–2.7)
3 (n = 29)	39.4 (27.9–43.3)	2.8 (2.3–4.1)
4 (n = 29)	56.3 (34.5–63.2)	4.4 (3.2–6.2)
5 (*n* = 29)	73.1 (44.5–80.9)	5.6 (3.9–7.8)
8 (n = 27)	101.2 (68.1–125.7)	8.9 (5.3–12.7)
15 (*n* = 23)	149.3 (88.8–152.3)	12.4 (8.7–13.3)
22 (*n* = 17)	150.1 (139.8–156.2)	14.0 (12.2–19.0)
29 (*n* = 12)	145.4 (127.7–153.2)	17.2 (11.9–18.6)
36 (*n* = 10)	149.5 (125.7–154.1)	18.4 (14.8–19.1)

The data are expressed as median (interquartile range)

### The effect of a changing intestinal oxygenation after feeding on cerebral oxygenation

Clustering all feeds observed, for all instances that the postprandial r_int_SO_2_ increased, the median postprandial increase was 7% (range 1–41, *n* = 21, 42 measurements) 10 to 30 min and 11% (range 1–41, *n* = 22, 40 measurements) 30 to 60 min after feeding, respectively. For all instances that the postprandial r_c_SO_2_ decreased, median postprandial decrease was − 5% (range − 22 to − 1, *n* = 29, 77 measurements) 10 to 30 min and − 4% (range − 31 to − 1, n = 29, 82 measurements) 30 to 60 min after feeding, respectively. We did not find an association between an increasing postprandial r_int_SO_2_ and a decreasing postprandial r_c_SO_2_. We did, however, find that the absence of an increasing postprandial r_int_SO_2_ was significantly associated with a 3.6 times higher odds ratio for a decreasing r_c_SO_2_ 10 to 30 min (95% CI, 1.5–8.9, *P* = <.01) and a 3.0 times higher odds ratio for a decreasing r_c_SO_2_ 30 to 60 min (95% CI, 1.2–7.3, *P* = .02) after feeding. Preprandial and postprandial r_c_SO_2_ (and cFTOE) values are presented in Table 2.

### Infants with and without the development of gastrointestinal complications

**Fig. 4:**
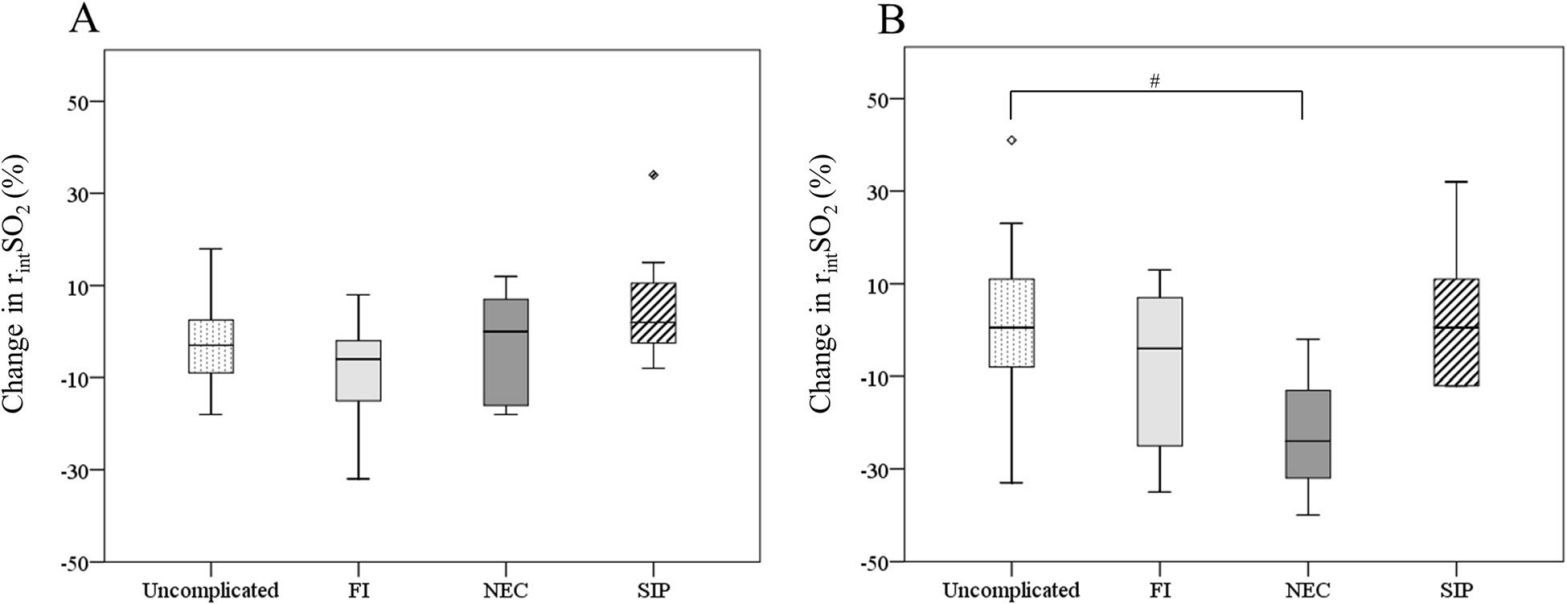
Postprandial change in r_int_SO_2_ values in infants with and without abdominal complications The boxes represent the change in r_int_SO_2_ values of the clustered data from the first two postnatal weeks between the 25th and 75th centiles (interquartile range) between baseline and 10–30 min after feeding (**a**) and between baseline and 30–60 min after feeding (**b**) for infants without abdominal complications (uncomplicated), infants who developed feeding intolerance (FI), necrotizing enterocolitis (NEC), and a spontaneous intestinal perforation (SIP); the whiskers represent the range of the values with the exception of outliers. Outliers are represented by the circles and diamonds, defined as values between 1.5 interquartile range and 3 interquartile ranges from the end of a box. ^#^ *<* .10

Seven infants developed FI (24%), three infants developed NEC (10%), and two (7%) infants developed SIP. We did not find a change in r_int_SO_2_ 10–30 min after feeding between infants who developed NEC and infants who did not develop a GI complication during the first two postnatal weeks. The infants who developed NEC, however, tended to have a decreasing r_int_SO_2_ 30–60 min after enteral feeding compared to infants without GI complications (− 24% vs. 1%, *P* = .06) during the first two postnatal weeks (Fig. 4). There was no change in r_int_SO_2_ 10–30 min and 30–60 min after feeding between infants who developed FI and infant without GI complications, and between infants who developed SIP and infants without GI complications (Fig. 4).

## Discussion

We demonstrated that in our group of preterm infants, born after approximately 28 weeks of gestation, a postprandial increase of intestinal oxygen saturation does occur, albeit at group level only in the fifth week after birth or in infants of a relatively older corrected gestational age. Furthermore, we showed that not a postprandial increase of intestinal oxygenation, but rather the absence thereof, was associated with a higher risk of a decrease of the cerebral oxygen saturation.

Our results suggest that during the first four weeks after birth at group level, intestinal perfusion does not exceed any potential increased oxygen consumption after enteral feeding. In the fifth week after birth the PMA of the remaining infants was 31.7 weeks. We assume that during this period the postprandial effect of feeding on the intestinal rSO_2_ can be explained by an increasing PMA rather than PNA, because we demonstrated that infants with a PMA ≥ 32 weeks have an increased postprandial r_int_SO_2_. In addition, by this time the remaining infants received relatively greater feeding volumes, which we demonstrated to be another important factor to elicit postprandial hyperaemia. Previous reports showed increased postprandial intestinal oxygen saturation using NIRS [2, 15, 16] or increased postprandial blood flow velocity of the SMA using Doppler measurements [3, 4], but these measurements were mainly done cross-sectionally, in fullterm and preterm infants with a corrected GA of at least 32 weeks, and were not assessed from birth onwards.

We offer several explanations for the fact that we did not find increased intestinal oxygen saturation after enteral bolus feeding during the first four weeks after birth and in the younger infants with a PMA < 32 weeks based on the principle that intestinal oxygenation consists of a balance between oxygen supply and consumption [15]. First, it may be that neither intestinal oxygen supply nor oxygen consumption changes in very preterm infants after enteral feeding because of intestinal immaturity on account of the fact that intestinal maturation is an ongoing process up to 33 to 34 weeks of gestation, and even beyond [27].

Besides intestinal immaturity, the low feeding volumes received during the first weeks after birth, especially in the youngest infants, may only result in a limited increase of intestinal metabolism and perfusion. Previous reports on animal models demonstrated a dose-dependent hyperaemic intestinal response after feeding [28, 29]. In addition, previous studies that reported increased intestinal perfusion after feeding were performed in preterm infants who tolerated feeding volumes of 100 mL/kg/day [2, 15–17]. We confirmed that indeed increased feeding volumes were associated with a higher chance of increasing intestinal saturation after feeding.

Another, but perhaps less likely explanation for not finding any change in intestinal oxygenation after feeding in the youngest infants, may come from a potentially perfect balance between oxygen supply and oxygen consumption. It may be that both increase equally after enteral feeding. One would, however, sooner expect such perfect harmony in the more mature infants.

Finally, several perinatal conditions may have influenced our findings. In comparison to populations reported on previously, our study population consisted of a relatively large proportion of infants who had a hemodynamically significant PDA. It has been demonstrated that preterm infants with large PDAs show a very slight increase of SMA blood flow velocities one hour after enteral bolus feeding compared to preterm infants without a PDA or a small or moderate PDA [30]. Therefore, in our study, the relative large proportion of infants with a PDA might have contributed to a lack of postprandial r_int_SO_2_ increase at group level. Additionally, other perinatal morbidities, (that is being born small for gestational age or anaemia, Table 1) may also have contributed to our results. Two recent studies demonstrated a lack of increase, or even a decrease, in postprandial r_int_SO_2_ in a group of anaemic preterm infants and in preterm infants who showed fetal signs of intrauterine growth restriction [31, 32]. Martini et al. showed that preterm infants with abnormal prenatal umbilical Doppler measurements lack any effect of the first enteral feeding on r_int_SO_2_ [29]. The results of these studies suggest that the intestinal response to enteral feeds is complex and that it is influenced by intestinal immaturity as well as intestinal condition and other perinatal factors [31, 32]. The hemoglobin levels in our study population decreased over time during the five weeks after birth, while we demonstrated that the r_int_SO_2_ increased after enteral feeding in the fifth week after birth. We therefore speculate that the maturing process of the intestine after birth and the greater feeding volumes have a larger contribution on the change in intestinal oxygen saturation after enteral feeding and attenuate the effect of the level of hemoglobin. Unfortunately, we were unable to perform subanalyses to address these issues on account of the size of our sample.

We did not observe an association between postprandially increased intestinal oxygen saturation and postprandially decreased cerebral oxygen saturation. On the contrary, we did find an association between a lack of a postprandial increase of the intestinal rSO_2_ and the risk of a decreased cerebral rSO_2_. We hypothesize that the infants with a lower PMA, who more often seemed to lack an adequate intestinal response, might have a less adequate cerebrovascular autoregulation and thus are at risk of compromised cerebral perfusion. A decrease of the cerebral oxygen saturation might indicate a decreased systemic circulation. One of the reasons for a decreased systemic circulation might be a lower cardiac output, but this might also be due to changes in blood pressure or redistribution of blood flow to other vital organs that temporarily have an increased metabolic demand. As the cerebral oxygen saturation is not a measure for cardiac output, assumptions concerning a decrease in cardiac output, or to what extent the cardiac output might have changed, cannot be made. Previously, stable cerebral oxygen saturation values were reported in studies that evaluated the effect of enteral feeding on rSO_2_ in preterm infants [2, 16, 33, 34]. Combining these results with our results suggests that cerebral saturation is not compromised when intestinal perfusion increases after enteral bolus feeding, possibly on account of adequate cerebrovascular autoregulation, but that this may be age-dependent.

In our study, only a few infants developed NEC. The infants who subsequently developed NEC tended to have a decreasing intestinal oxygen saturation 30–60 min after bolus feeding during the first two postnatal weeks, whereas infants without GI complications, FI, and SIP did not. As a result of the very small number of infants, these results have to be carefully interpreted, and conclusions cannot be made based on these results, which require further investigation in a larger cohort.

An important strength of this exploratory study is the longitudinal design that created the opportunity to address the age-dependent component on the effect of enteral feeding on intestinal perfusion. Nevertheless, we also recognise several limitations to our study. The first limitation was the relative small population studied. Therefore we could not analyse the influence of comorbidities on intestinal perfusion after enteral feeding. Neither could we stratify the study cohort on the basis of feeding intervals or feeding type, nor could we perform multivariable regression analyses to test for possible confounding factors. Despite the fact that we performed several tests we chose not to correct for multiple testing, because we considered this observation to be exploratory and hypothesis generating. Another limitation concerns validity issues using NIRS to assess intestinal oxygenation. Movement of the gut, abdominal gasses, and stools could influence the signal because of absorption changes of the near-infrared light which is path-length dependent [6, 14, 31, 32, 35]. Additionally, standard limits of intestinal oxygen saturation are not yet established on account of the wide intervariability and intravariability of intestinal rSO_2_ values [14, 16]. Finally, we clustered our data to determine whether the effect of enteral feeding on the intestinal rSO_2_ depends on PMA. Therefore the contribution of measurements per infant was unequally distributed and we might have underestimated or overestimated our results. Nevertheless, to our knowledge, this is the first longitudinal study demonstrating that enteral feeding only affects intestinal oxygen saturation after weeks, or when infants have reached 32 weeks PMA, and larger volumes of feeds.

## Conclusions

Our results suggest that postprandial intestinal hyperaemia does only occur at group level from the fifth week after birth or in infants with relatively older corrected gestational ages receiving a greater amount of enteral feeding. In addition, we showed that postprandial intestinal hyperaemia is not associated with compromised cerebral perfusion. Our study provides more insight into the intestinal physiologic response to enteral feeding in preterm infants. A better understanding of this intestinal physiologic postprandial response might support clinicians in identifying infants at risk for the development of GI complications. This exploratory study, however, raises questions about when and why intestinal saturation does or does not increase after enteral bolus feeding in the early postnatal weeks of a preterm infant, and whether a decreasing intestinal perfusion after feeding may be associated with GI complications later on. Further study is required to address these issues. Moreover, larger studies addressing possible confounders on the intestinal haemodynamic response to enteral feeds, such as PDA and other perinatal morbidities, are needed.

## Data Availability

The data sets generated and analysed during this study are available from the corresponding author on reasonable request.

## Abbreviations

BW: Birth weight
cFTOE: cerebral fractional tissue oxygen extraction
CHD: Congenital heart disease
FEF: Full enteral feeding
FI: Feeding intolerance
FTOE: Fractional tissue oxygen extraction
GA: Gestational age
GI: Gastrointestinal
intFTOE: intestinal fractional tissue oxygen extraction
NEC: Necrotizing enterocolitis
NICU: Neonatal intensive care unit
NIRS: Near-infrared spectroscopy
PDA: Patent ductus arteriosus
PMA: Postmenstrual age
PNA: Postnatal age
r_c_SO_2_: Regional cerebral tissue oxygen saturation
r_int_SO_2_: Regional intestinal oxygen saturation
rSO_2_: Regional tissue oxygen saturation
SE: Standard error of the mean
SMA: **S**uperior mesenteric artery
SpO_2_: Arterial oxygen saturation
